# Diabetic Mice Spleen Vulnerability Contributes to Decreased Persistence of Antibody Production after SARS-CoV-2 Vaccine

**DOI:** 10.3390/ijms251910379

**Published:** 2024-09-26

**Authors:** Yara Atef, Tomoya Ito, Akitsu Masuda, Yuri Kato, Akiyuki Nishimura, Yasunari Kanda, Jun Kunisawa, Takahiro Kusakabe, Motohiro Nishida

**Affiliations:** 1 Department of Physiology, Graduate School of Pharmaceutical Sciences, Kyushu University, Fukuoka 812-8582, Japan; 2 Laboratory of Creative Science for Insect Industries, Graduate School of Pharmaceutical Sciences, Kyushu University, Fukuoka 819-0395, Japan; a.masuda@agr.kyushu-u.ac.jp; 3 National Institute for Physiological Sciences (NIPS), National Institutes of Natural Sciences (NINS), Okazaki 444-8787, Japan; aki@nips.ac.jp; 4 Exploratory Research Center on Life and Living Systems (ExCELLS), National Institutes of Natural Sciences (NINS), Okazaki 444-8787, Japan; 5 Department of Physiological Sciences, School of Life Science, The Graduate University for Advanced Studies (SOKENDAI), Okazaki 444-8787, Japan; 6 Division of Pharmacology, National Institute of Health Sciences, Kawasaki 210-9501, Japan; kanda@nihs.go.jp; 7 Laboratory of Vaccine Materials, Microbial Research Center for Health and Medicine, National Institutes of Biomedical Innovation, Health and Nutrition, Osaka 567-0085, Japan; 8 Laboratory of Gut Environmental System, Microbial Research Center for Health and Medicine, National Institutes of Biomedical Innovation, Health and Nutrition, Osaka 567-0085, Japan; 9 Laboratory of Insect Genome Science, Kyushu University Graduate School of Bioresource and Bioenvironmental Sciences, Fukuoka 819-0395, Japan; kusakabe@agr.kyushu-u.ac.jp

**Keywords:** diabetes, obesity, COVID-19, SARS-CoV-2, S-protein, vaccination, adjuvant optimization, spleen

## Abstract

During the COVID-19 pandemic, diabetic and obese patients experienced higher rates of hospital admissions, severe illness, and mortality. However, vaccinations failed to provide those vulnerable populations the same level of protection against COVID-19 severity as those without diabetic and obese phenotypes. Our study aimed to investigate how diabetes mellitus (DM) impacts the immune response following vaccination including the artificially designed trimeric SARS-CoV-2 spike (S)-protein. By using two diabetic mouse models, ob/ob mice (obese, hyperglycemic, and insulin-resistant) and STZ-treated mice (insulin-deficient and hyperglycemic), we observed a significant reduction in S-protein-specific IgG antibody titer post-vaccination in both diabetic models compared to wild-type (WT) mice. Both diabetic mouse models exhibited significant abnormalities in spleen tissue, including marked reductions in splenic weight and the size of the white pulp regions. Furthermore, the splenic T-cell and B-cell zones were notably diminished, suggesting an underlying immune dysfunction that could contribute to impaired antibody production. Notably, vaccination with the S-protein, when paired with an optimal adjuvant, did not exacerbate diabetic cardiomyopathy, blood glucose levels, or liver function, providing reassurance about the vaccine′s safety. These findings offer valuable insights into potential mechanisms responsible for the decreased persistence of antibody production in diabetic patients.

## 1. Introduction

The prevalence of diabetes mellitus (DM) and obesity has been rising across the globe. Among American adults, the incidence rates of DM and obesity are 14.8% and 41.9%, respectively [1]. Population-based observational studies have shown a strong correlation between increased infection risks and both type 1 and type 2 DM, leading to higher rates of morbidity and mortality [2]. Similarly, after the emergence of coronavirus disease 2019 (COVID-19) as a global pandemic, individuals with DM and obesity faced an elevated risk. Case series revealed that approximately 30–40% of hospitalized COVID-19 patients had either type 1 or type 2 diabetes and that diabetic individuals faced a 100–250% higher risk of morbidity and mortality compared to those without diabetes [3]. Moreover, obesity in COVID-19 diabetic patients was significantly associated with higher odds of severe pneumonia and increased need for in-hospital oxygen therapy [4].

COVID-19, caused by severe acute respiratory syndrome coronavirus-2 (SARS-CoV-2), causes symptoms ranging from mild illness to severe lung injury, organ failure, and death, specifically for patients with other comorbidities [5,6,7]. The SARS-CoV-2 infection caused significant global mortality and morbidity, with no treatment options initially available. Vaccination became the most effective intervention, particularly for protecting vulnerable populations and reducing the disease burden.

The COVID-19 vaccine has indeed played a pivotal role in our fight against the pandemic. With more than 13.5 billion doses of vaccines administered worldwide, we may consider that 70% of the world’s population has received at least one dose of a COVID-19 vaccine [8]. In international studies involving large sectors of the population, SARS-CoV-2 spike mRNA vaccines have been found to be safe. The COVID-19 vaccine has effectively reduced prevalence, hospitalization rates, mortality, and morbidity [9]. However, some studies have reported that type 2 DM (T2DM) patients who received COVID-19 vaccines showed significantly decreased antibody responses compared to healthy individuals and that T2DM patients with good glycemic control (HbA1c one-year mean <7%) exhibited a higher virus-neutralizing antibody capacity compared to those with poor control (HbA1c one-year mean ≥7%) [10,11]. Furthermore, the COVID-19 vaccine-induced antibody protection declined more rapidly in individuals with severe obesity [12,13]. Well-controlled blood glucose levels prior to vaccination were associated with stronger spike antibody binding in type 1 DM (T1DM), indicating that the glucose profile plays a crucial role in vaccination efficacy [14]. Moreover, vaccinated T1DM patients failed to exhibit any increase in the SARS-CoV-2-specific cytotoxic response, unlike the robust increase observed in all non-diabetic subjects [15]. These findings suggest that vaccine-induced immunological response declines faster in diabetic and obese individuals.

Commercially available vaccines contain different adjuvants, components that enhance the immune response, tailored by each manufacturer. Aluminum hydroxide (Alum) is the most used adjuvant in vaccines, including some COVID-19 vaccines, for its safety and effectiveness in clinical trials [16]. MF-59, an oil-in-water emulsion-based adjuvant, has been shown to enhance the immunogenicity of Middle East Respiratory Syndrome (MERS) vaccines while maintaining an excellent safety profile [17]. Lipid A, a component of the lipopolysaccharides (LPSs) of the Gram-negative bacteria outer membrane, was shown to enhance the immunogenicity of mRNA vaccines [18]. In addition, MPL, a derivative of Lipid A, has been previously combined with other adjuvants to increase the protective efficacy of vaccines [19].

In our study, we intend to examine how both DM and obesity influence the immunological response to an S-protein mixture vaccination. To fill existing gaps in the knowledge, we will first identify the optimal adjuvant for the S-protein antigen mixture to maximize antibody production. Subsequently, we will administer this optimized vaccine to two distinct diabetic mouse models: obese, hyperglycemic, and insulin-resistant ob/ob mice [20] and insulin-deficient, hyperglycemic Streptozotocin (STZ)-induced diabetic mice [21].

## 2. Results

### 2.1. MF-59 Adjuvant with S-Protein Antigen Mixture Yields the Highest IgG Antibody Titer

**Figure 1:**
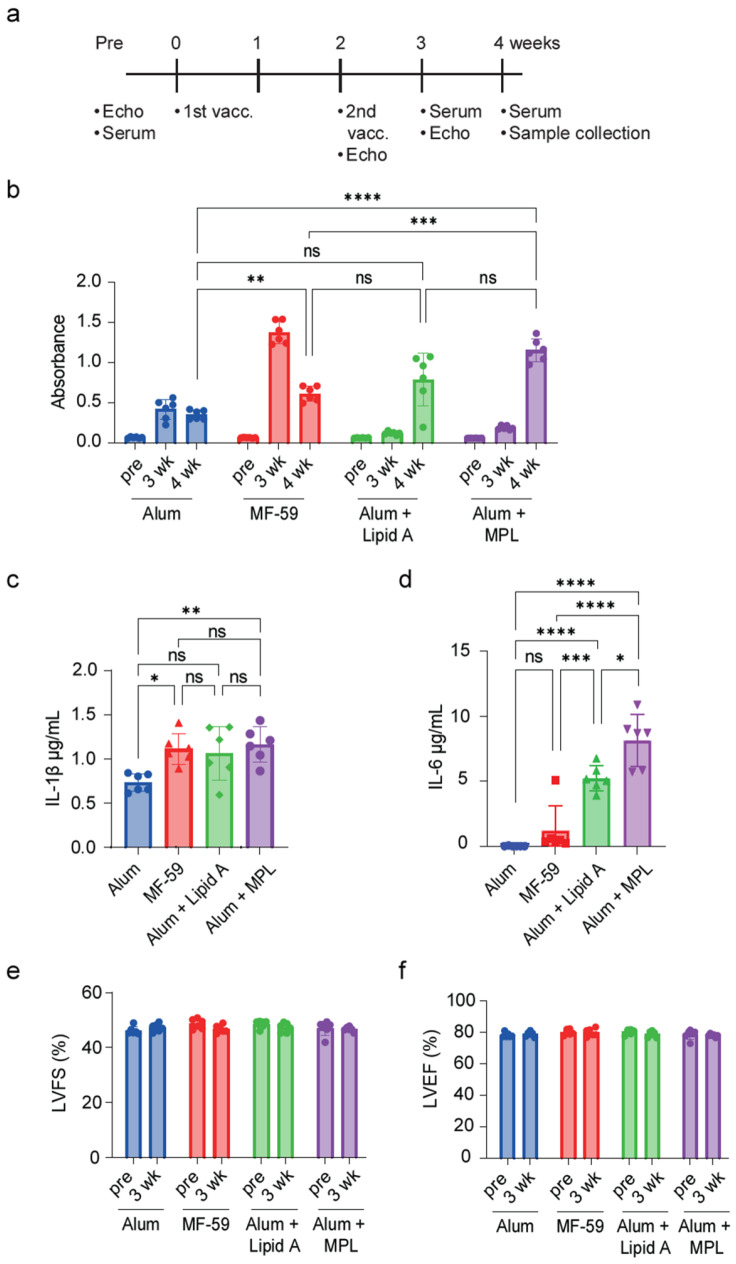
Investigation of the optimal adjuvant for the S-protein antigen mixture. (**a**) Schematic of study design for optimal adjuvants. (**b**) ELISA quantification of serum IgG antibody titer. (**c**,**d**) ELISA quantification of serum IL-1β (**c**) and IL-6 (**d**). *n* = 6 mice in each group. (**e**,**f**) Quantification of 4D-mode LV fractional shortening (LVFS) (**e**) and LV ejection fraction (LVEF) (**f**). Data are shown as mean ± SEM. Significance was determined using one-way ANOVA followed by Tukey’s post hoc test (**e**,**f**) or two-way ANOVA followed by Bonferroni comparison test (**b**–**d**). ns: non-significant, * *p* < 0.05, ** *p* < 0.01, *** *p* < 0.001, and **** *p* < 0.0001.

Initially, our objective was to optimize the S-protein mixture (D614G variant and Omicron BA.1 variant) and adjuvant combination. We tested four adjuvants: Alum, MF-59, Alum + Lipid A, and Alum + MPL (Figure 1a). MF-59 and Alum + MPL showed significant robustness in the S-protein-specific IgG antibody titer when compared to the Alum group at the 4-week mark (Figure 1b). However, Alum + MPL and Alum + Lipid A were associated with increased serum inflammatory cytokine levels of IL-1β and IL-6 (Figure 1c,d). Consequently, MF-59 was selected as the most suitable adjuvant. Furthermore, since COVID-19 vaccines were previously reported to cause cardiovascular adverse effects [22,23], echocardiography was used to assess cardiac functions. None of these adjuvants, when combined with the S-protein mixture, demonstrated a significant impact on cardiac functions (Figure 1e,f). Subsequent experiments were conducted to assess the impact of the S-protein mixture and Mf-59 adjuvant on two diabetic models: ob/ob mice and STZ-treated mice.

### 2.2. S-Protein Has No Impact on Hyperglycemic Diabetic Cardiomyopathy

**Figure 2:**
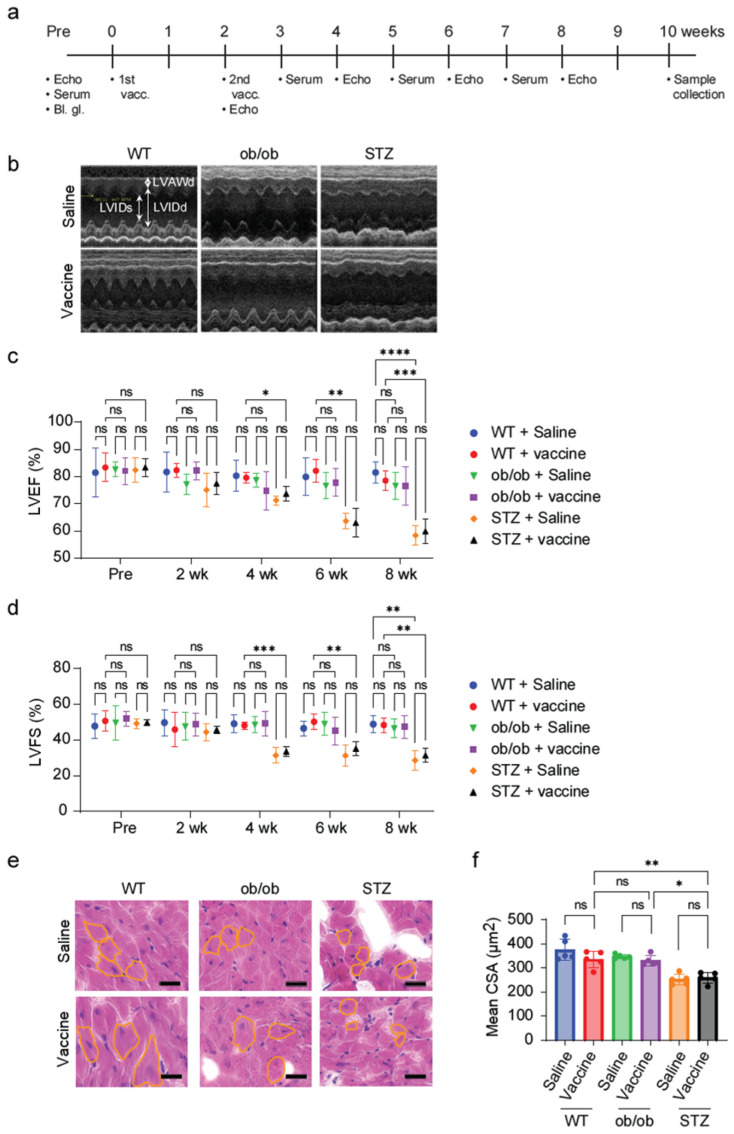
S-protein vaccine administration does not exacerbate hyperglycemic diabetic cardiomyopathy. (**a**) Schematic of study design for analyzing cardiac functions in two diabetic mouse models. (**b**) Images representing LV echocardiography in M-mode at 8 weeks post-vaccination. (**c**) Quantitative analysis of LV ejection fraction (LVEF) in 4D mode. (**d**) Quantitative analysis of LV shortening (LVFS) in 4D mode. (**e**) Images representing myocardium tissue sections stained with hematoxylin and eosin. Orange lines represent the cardiomyocytes cross-sectional areas (**f**) Quantitative analysis of the myocardium cross-sectional area (CSA) quantified from the HE staining in each group using ImageJ V1.54g. *n* = 5 mice in each group. Scale bar, 20 μm. Data are shown as mean ± SEM. Significance was determined using two-way ANOVA followed by Bonferroni comparison test (**c**,**d**) or one-way ANOVA followed by Tukey’s post hoc test (**f**). ns: non-significant, * *p* < 0.05, ** *p* < 0.01, *** *p* < 0.001, and **** *p* < 0.0001. (Bl. Gl. (blood glucose), vacc. (vaccination), LV (left ventricle), LVEF (left ventricle ejection fraction), LVFS (left ventricle fractional shortening), and CSA (cross-sectional area)).

Administering the S-protein-MF59 vaccine mixture to the diabetic mouse models, STZ and ob/ob, which were known to develop diabetic cardiomyopathy, raised concerns that the vaccine might exacerbate cardiac dysfunction [24]. Therefore, an echocardiography was conducted before vaccination and every other week thereafter to assess heart function (Figure 2a). In the saline-administered groups, the WT and ob/ob mice groups showed no decrease in LVEF (left ventricular ejection fraction) and LVFS (left ventricular fractional shortening), whereas the STZ-treated mice showed a significant reduction in LVEF and LVFS at 4 weeks and thereafter (Figure 2b–d). This result suggested that the STZ treatment itself caused cardiac dysfunction, i.e., hyperglycemic diabetic cardiomyopathy. When comparing vaccinated mice to their non-vaccinated counterparts in the WT and ob/ob groups, no significant difference in cardiac functions were observed. Additionally, administration of the COVID-19 vaccine to the STZ mice did not lead to any further deteriorating cardiac functions that was observed in the saline STZ-treated group.

To further explore the reduced ejection fraction in the STZ-treated groups, histological examination with HE staining was performed and the myocardial cross-sectional area (CSA) was calculated. The myocardial CSA was found to be reduced in both the vaccinated and saline STZ-treated groups compared to the WT and ob/ob group. However, there was no significant difference between the vaccinated and non-vaccinated mice in each diabetic model (Figure 2e,f). Taken together, the S-protein vaccine has no additional declining effect on the heart function in insulin-resistant and hyperglycemic diabetic mice.

### 2.3. S-Protein Vaccine Has No Effect on Blood Glucose and Serum Insulin in Diabetic Mice

**Figure 3:**
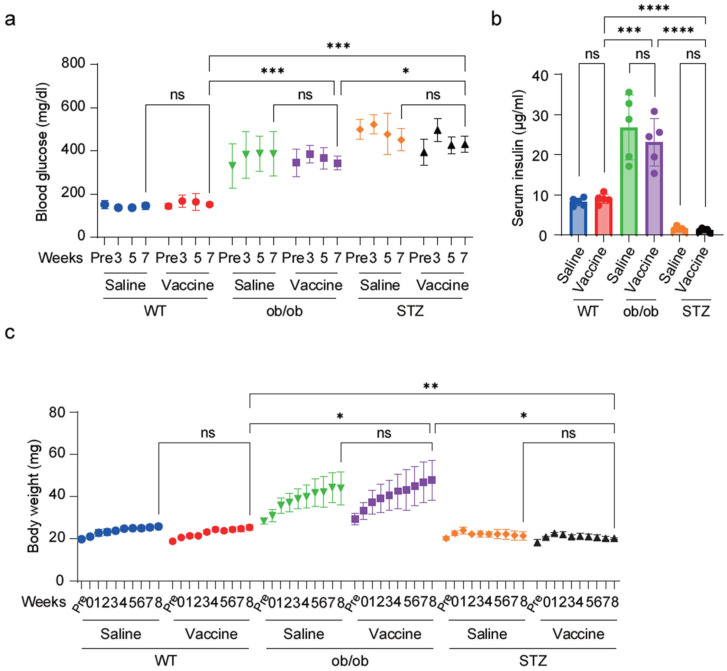
S-protein vaccine does not influence blood glucose and serum insulin. (**a**) Quantification of blood glucose level before and at 3, 5, and 7 weeks after vaccination. (**b**) Serum insulin levels at 10 weeks post-vaccination. (**c**) Time course of weekly body weight. *n* = 5 mice in each group. Data are shown as mean ± SEM. Significance was determined using one-way ANOVA followed by Tukey’s post hoc test (**b**) or two-way ANOVA followed by Bonferroni comparison test (**a**,**c**). ns: non-significant, * *p* < 0.05, ** *p* < 0.01, *** *p* < 0.001, and **** *p* < 0.0001.

We then assessed the impact on blood glucose, insulin levels, and body weight. The ob/ob and STZ-treated mice displayed a diabetic profile with elevated blood glucose levels (Figure 3a).

The ob/ob mice exhibited elevated serum insulin levels compared to both the WT and STZ groups, while the STZ treatments resulted in diminished serum insulin levels (Figure 3b). The ob/ob mice also showed a significant increase in body weight when compared to the WT and STZ-treated mice, whereas the STZ-treated mice showed decreased body weight relative to the other groups (Figure 3c). No significant differences in the blood glucose, serum insulin levels, or body weight were observed between the vaccinated and saline-treated mice within each study model.

### 2.4. S-Protein Vaccine Has No Effect on Liver Function in Diabetic Mice

**Figure 4:**
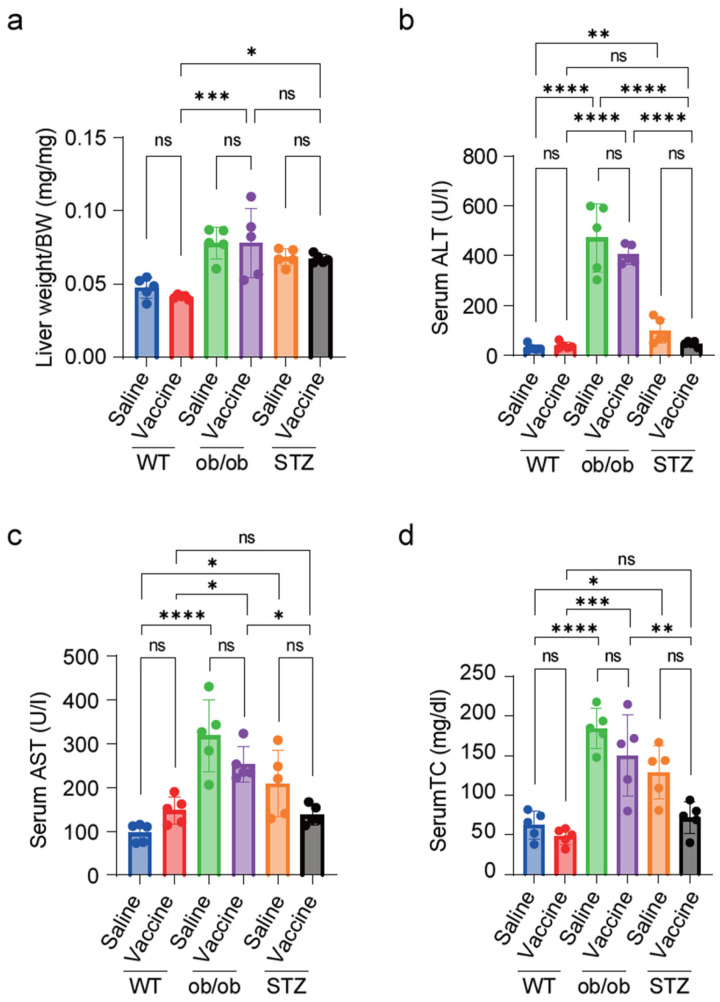
S-protein vaccine might possess a hepatoprotective effect in diabetic models. (**a**) Liver weight/body weight ratio. (**b**–**d**) Quantification of serum ALT (**b**), serum AST (**c**), and serum total cholesterol levels (**d**). Saline-treated and vaccinated WT, STZ-treated mice and ob/ob mice serum liver enzymes and total cholesterol (TC) levels were measured at 10 weeks after vaccination. *n* = 5 mice in each group. Data are shown as mean ± SEM. Significance was determined using one-way ANOVA followed by Tukey’s post hoc test. ns: non-significant, * *p* < 0.05, ** *p* < 0.01, *** *p* < 0.001, and **** *p* < 0.0001.

In the current study, the ob/ob and STZ-treated mice had a significant elevation in the liver weight/body weight ratio (Figure 4a). Both the ob/ob mice and STZ-treated mice exhibited elevated levels of ALT and AST, indicating liver damage. Notably, the ob/ob mice had higher liver enzyme levels than the STZ mice. The S-protein-vaccinated groups showed a tendency to reduce liver enzymes; however, this reduction was not statistically significant (Figure 4b,c).

We then assessed the total cholesterol (TC) levels in the serum. Both the ob/ob and STZ mice showed marked elevation in the serum TC levels (Figure 4d). Interestingly, the STZ-treated mice post-vaccination were found to have no significant difference in ALT, AST, and total cholesterol levels when compared to the vaccinated WT mice.

### 2.5. Vaccinated Diabetic Mice Decreased Persistence of S-Protein-Specific IgG Antibody Production

**Figure 5:**
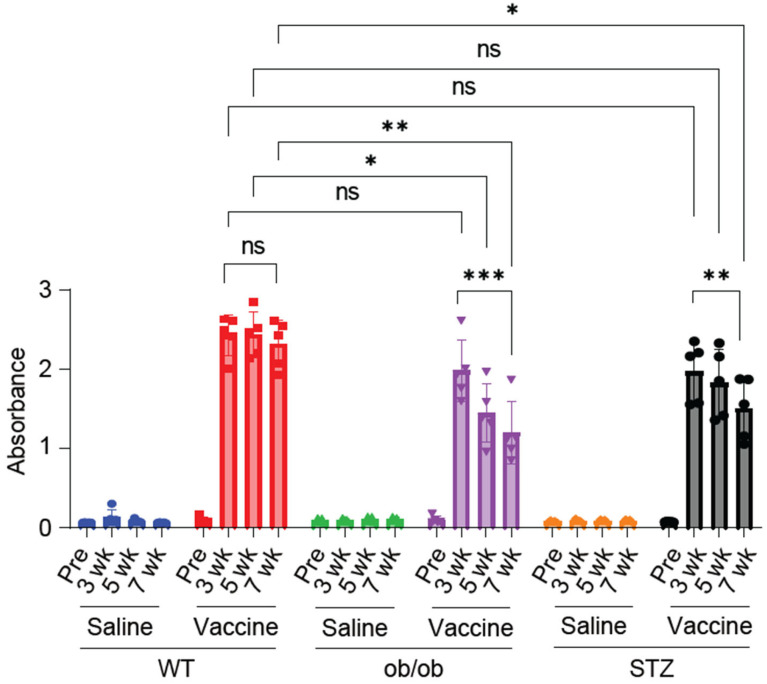
Vaccinated diabetic mice showed a marked decline in S-protein IgG antibody titer. Measurement of serum S-protein IgG antibody titer before and at 3, 5, and 7 weeks post-vaccination. *n* = 5 mice in each group. Data are shown as mean ± SEM. Significance was determined using two-way ANOVA followed by Bonferroni comparison test. ns: non-significant, * *p* < 0.05 and ** *p* < 0.01, and *** *p* < 0.001.

To assess the variations in IgG antibody production across the study models, we measured the serum levels of the S-protein-specific IgG antibodies using ELISA. The IgG levels were assessed before and after vaccine administration at 3, 5, and 7 weeks. All the vaccinated groups exhibited robust IgG antibody levels when compared to the saline-treated groups. At 3 weeks, no significant differences in antibody production were observed among the WT, ob/ob, and STZ vaccinated mice. By 5 weeks, the ob/ob mice showed a significant reduction in IgG antibody levels compared to the vaccinated WT mice, while the STZ-treated mice did not exhibit a significant difference compared to the WT group. At 7 weeks, both the vaccinated STZ-treated and ob/ob mice displayed reduced IgG antibody levels compared to the vaccinated WT group. Notably, we analyzed the variations within each study model over the course of the experiment. While the WT mice showed no significant differences between the 3- and 7-week mark, both diabetic models demonstrated a significant reduction in the IgG antibody level at the 7-week compared to the 3-week mark. (Figure 5).

### 2.6. Splenic White Pulp Is Reduced in Diabetic Mice

**Figure 6:**
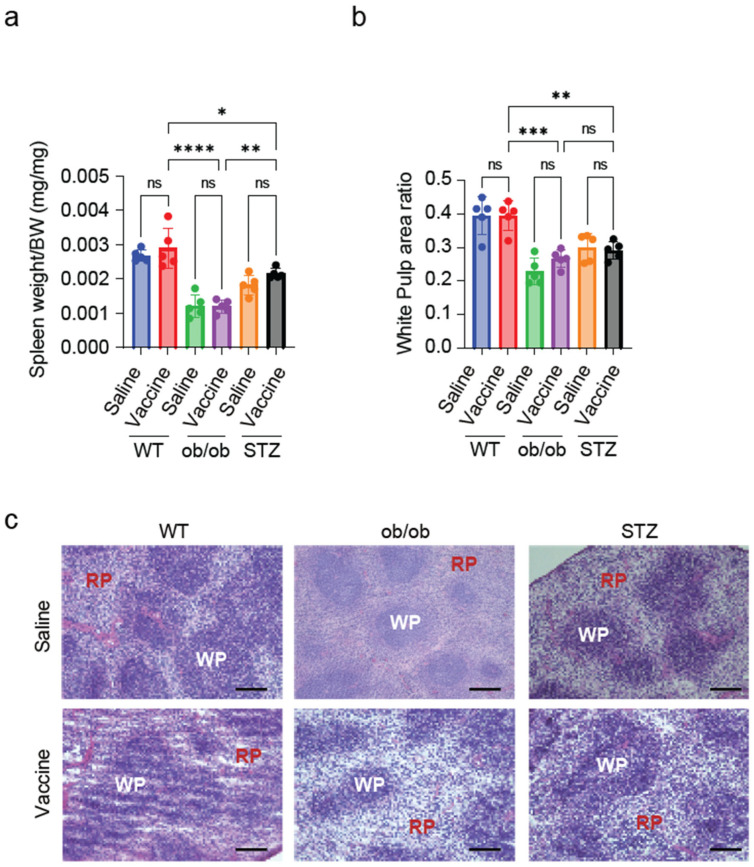
Vaccinated diabetic mice showed marked decline in the spleen. (**a**) Spleen weight/body weight ratio. (**b**) Quantitative results of spleen–white pulp area ratio. (**c**) Representative images of spleen tissue stained with HE showing WP (white pulp) and RP (red pulp) regions. *n* = 5 mice in each group. Scale bar, 200 μm. Data are shown as mean ± SEM. Significance was determined using one-way ANOVA followed by Tukey’s post hoc test. ns: non-significant, * *p* < 0.05, ** *p* < 0.01, *** *p* < 0.001, and **** *p* < 0.0001.

To further investigate the potential mechanisms underlying the reduced antibody production, we analyzed spleen tissue in the study models. The spleen weight/body weight ratio was significantly reduced in both diabetic model groups (Figure 6a). The splenic structure was then analyzed by examining tissue sections stained with HE. A significant reduction in the white pulp area was observed in the vaccinated ob/ob mice and STZ-treated mice when compared to the vaccinated WT mice (Supplementary Figure S1 and Figure 6b,c). The extent of the reduction in white pulp area in the ob/ob and STZ mice correlates with the decrease in IgG antibody production in these groups following S-protein vaccination.

### 2.7. Diabetic Mice Showed a Reduction in T-Cell and B-Cell Zones in the Splenic Tissue

**Figure 7:**
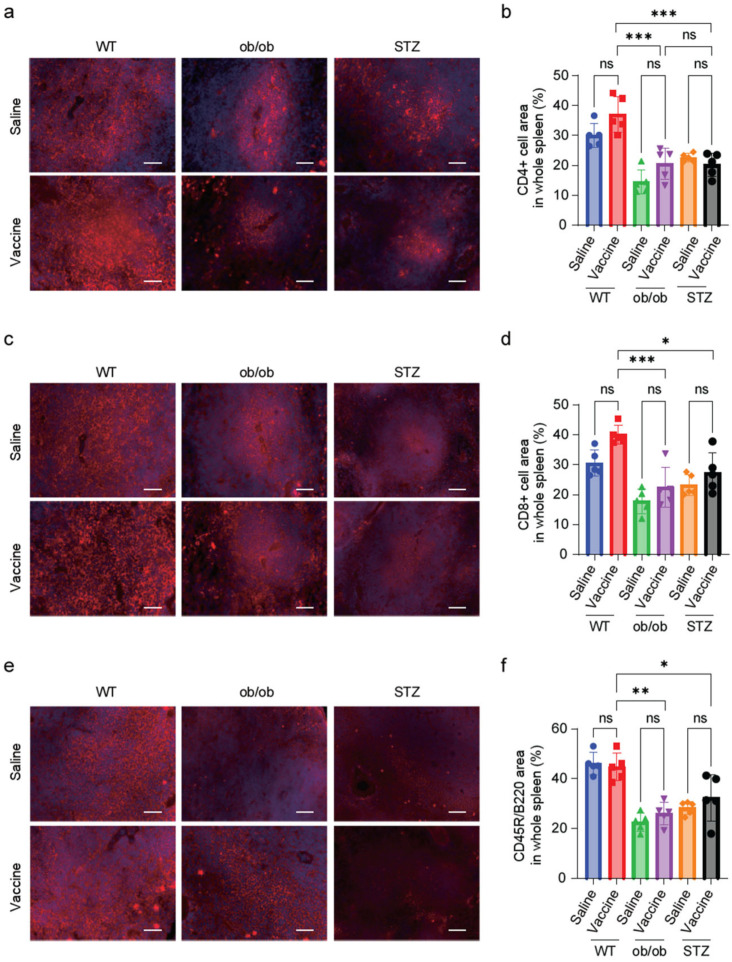
Splenic T-cell and B-cell zones showed a reduction in diabetic mice. (**a**,**c**,**e**) Representative images of IHC-stained spleen tissue showing CD4 (red) to identify T cells (**a**), CD8 (red) to identify T cells (**c**), and CD45R/B220 (red) to identify B cells (**e**), nuclei were stained with DAPI (blue). (**b**,**d**,**f**) Quantification of positive cell area for splenic CD4 (**b**), CD8 (**d**), and CD45R/B220 (**f**). *n* = 5 mice in each group. Scale bar, 100 μm. Data are shown as mean ± SEM. Significance was determined using one-way ANOVA followed by Tukey’s post hoc test. ns: non-significant, * *p* < 0.05, ** *p* < 0.01, and *** *p* < 0.001.

T and B lymphocytes, which are responsible for antibody production, reside in the T-cell and B-cell zones in the spleen, respectively [25]. To investigate whether hyperglycemia affects the T- and B-cell zones in the spleen, spleen tissue sections were stained with CD4 and CD8 to visualize the T-cell zones and CD45R/B220 antibodies to identify the B-cell zones. Calculating the areas of CD4- and CD8-positive areas in whole spleen sections showed a marked reduction in the ob/ob mice and STZ-treated mice when compared to the WT groups (Supplementary Figure S2a,b and Figure 7a–d). Additionally, the B-cell zones were reduced in the diabetic mice groups when compared to WT (Supplementary Figure S2c and Figure 7e,f). The amount of reduction in the B-cell and T-cell zone areas in the ob/ob and STZ-treated mice correlated with the decrease in S-protein IgG antibody production.

## 3. Discussion

In the presented study, we demonstrated that diabetic mice—ob/ob mice (obese, hyperglycemic, and insulin-resistant) and STZ-treated mice (hyperglycemic and insulin-deficient)—exhibit spleen abnormalities that impair both humoral and cellular immunogenicity. As a result, these mice show reduced antibody production and diminished protection following two doses of the S-protein vaccine compared to the wild-type (WT) mice. This provides evidence supporting similar observations in human patients and establishes a model for investigating the relationship between metabolic diseases and vaccine responses.

In the beginning, we administered the S-protein antigen mixed with each of four different adjuvants separately in the different study groups. MF-59 was the one that appeared to boost the antibody titer the most while maintaining minimal cytokine induction and having no influence on cardiac functions. The MF-59 adjuvant was previously found to enhance the antibody and cellular responses of three protein-based COVID-19 vaccines compared to Alum [26].

The S-protein-MF-59 mixture was later administered to the two diabetic models: ob/ob mice and STZ-treated mice. Since cardiovascular diseases are the leading cause of death among diabetic patients, who are at a higher risk of heart function failure compared to non-diabetic individuals of the same age [27], echocardiography was performed to assess the cardiac function. In our study, STZ treatment significantly impaired the LVEF and LVFS, while vaccination with the S-protein did not lead to further deterioration in cardiac function. Moreover, vaccination had no impact on cardiac functions in the other study groups. The histological analysis supported these findings, showing that STZ-treated mice had reduced myocardial cell cross-sectional area, with no significant differences between the vaccinated and non-vaccinated mice. This suggests that an S-protein vaccine does not exacerbate cardiomyopathy.

The COVID-19 vaccine was linked to hyperglycemia in some cases [28,29], so blood glucose was sought to be measured every other week. Undoubtedly, there was marked elevation in the blood glucose levels of the ob/ob mice, attributed to their enlarged pancreatic cells and insulin resistance, as well as the STZ-treated mice, where STZ treatment selectively destroys pancreatic beta cells and leads to insulin deficiency [20,30].

However, no changes were noticed between the vaccinated and non-vaccinated mice of the same study model, suggesting that the S-protein vaccine has no influence on blood glucose in diabetes.

Non-vaccinated STZ-treated mice and ob/ob mice were known to exhibit diabetic liver disease and alterations in their lipid profile [20,31], which aligns with our findings that both models show elevated liver enzymes and total cholesterol levels when compared to the WT group. The vaccinated WT, ob/ob, and STZ-treated mice exhibited no further elevation in liver enzymes post-vaccination. However, the vaccinated STZ-treated mice showed no significant difference in AST and TC when compared to the WT vaccinated mice. This finding suggests that the S-protein vaccine may have a hepatoprotective effect, supporting previous evidence that two doses of the COVID-19 vaccine was able to reduce the incidence of liver injury and related outcomes in infected patients [32]. This reinforces the safety profile of S-protein vaccine administration, particularly for individuals with vulnerable liver conditions.

To assess differences in humoral immunity after vaccination between study models, the S-protein-specific IgG antibody levels were measured. At 3 weeks, antibody production was similar across the vaccinated WT, ob/ob, and STZ-treated mice. However, by 5 weeks, the ob/ob mice exhibited a significant reduction in IgG antibody levels compared to the vaccinated WT group, while the STZ-treated mice showed no significant difference from the WT mice. Finally, by 7 weeks, both the vaccinated STZ-treated and ob/ob mice exhibited reduced IgG antibody levels compared to the vaccinated WT group.

The interesting difference in the IgG antibody levels post-vaccination between the WT mice and the two diabetic models pushed toward further research in the splenic tissue. The spleen, a crucial organ in the immune system, has two primary tissue types, red pulp and white pulp, each playing distinct roles in blood filtration and immune function [25]. The red pulp filters the blood and contains macrophages that engulf and digest cellular debris and pathogens. The white pulp, on the other hand, is crucial for initiating immune responses. This region contains the T- and B-cell zones, where essential lymphocytes (T and B cells) interact and activate each other to facilitate antibody production [33]. In the presented work, the spleen index was found to be reduced in both the ob/ob and STZ-treated mice compared to the WT mice, indicating that diabetes and/or obesity may lead to splenic abnormalities. Moreover, when HE-stained splenic tissue sections were examined, and the white pulp-to-whole-spleen-area ratio was calculated, a significant reduction was observed in the white pulp area between the diabetic mice, ob/ob and STZ-treated, and WT mice. Previous studies have reported that diabetic and/or obese mice exhibit a reduction in spleen size. STZ treatment has been shown to decrease spleen size, while mice with diet-induced obesity (DIO) display a reduced spleen index [34,35].

Effective antibody production in the spleen’s white pulp relies on interactions between T and B cells. T cells play a key role in cell-mediated immunity. CD4+ T cells, also known as helper T cells, are crucial for activating B cells and other immune cells. CD8+ T cells, or cytotoxic T cells, are involved in directly killing infected or cancerous cells and are also present in the PALS [36]. In the presented work, CD4+- and CD8+-stained spleen tissue sections were examined and the CD4+ and CD8+ cell areas were calculated. A marked reduction in both the CD4+ and CD8+ cell areas was observed in the ob/ob and STZ-treated mouse models, indicating a reduction in T-cell zones.

Furthermore, B cells are crucial for antibody production. B cells recognize antigens, are activated by helper T cells, and differentiate into plasma and memory cells that secrete antibodies [33]. CD45R/B220, a marker expressed on B cells [37], indicates active B-cell zones in the spleen where antibody production occurs. Our study used immunohistochemical staining with the B-cell CD45R/B220 antibody on spleen sections and revealed an inhibition in the B-cell area in the ob/ob and STZ-treated mice when compared to the WT mice.

Unlike WT mice, both diabetic models exhibited marked reduction in the white pulp, T-cell, and B-cell regions, which is more likely linked to the decreased S-protein-specific IgG antibody levels observed between vaccinated WT mice and diabetic mice. These findings suggest that diabetes leads to impaired immunity and splenic abnormalities. By establishing this connection between diabetes and vaccine efficacy, our study paves the way for further investigations into the mechanisms behind reduced vaccine responses.

There are several mechanisms that explain impaired immunity in diabetes. High blood glucose levels increase the production of reactive oxygen species (ROS), which can damage immune cells, affect T-cell activation and B-cell differentiation, and thereby limit their response and impair their function [38]. Hyperglycemia is also linked to the formation of advanced glycation end-products (AGEs), which bind to receptors on immune cells, such as macrophages and T cells, further disrupting the immune response [39]. It also skews the balance of pro- and anti-inflammatory cytokines, often causing excessive inflammation, which reduces T-cell activation and B-cell antibody production [40]. Additionally, hyperglycemia impairs the ability of immune cells to migrate to infection sites and reduces phagocytic activity, making it harder for CD4 and CD8 T cells, as well as B cells, to coordinate an effective immune response [41].

In summary, unlike WT mice, both hyperglycemic obese insulin-resistant and hyperglycemic insulin-deficient diabetic models failed to maintain robust antibody production after receiving two doses of S-protein antigen mixture vaccine. Both diabetic models exhibited evident splenic abnormalities and reduced humoral and cellular immunity. These findings could provide insights into the possible mechanisms underlining the decreased persistence of antibody production in diabetic patients and push through the necessity of vaccine booster doses to diabetic individuals to achieve comparable immunological protection to that of the general population.

## 4. Materials and Methods

### 4.1. Animals

All the protocols using mice were reviewed and approved by the ethics committee nos. A24-163-0 and A23-371-0 at Kyushu University and carried out in accordance with the committee guidelines. The BALB/cCrSlc, wild-type (WT) C57BL/6, and ob/ob 6-week-old male mice were obtained from the Japan SLC, Inc. (Shizuoka, Japan).

The STZ (Cat# 191-15151, FUJIFILM Wako Pure Chemical Corporation, Osaka, Japan)-treated mice were administered a single intraperitoneal injection of STZ (180 mg/kg body weight) freshly prepared in citrate buffer (pH 4.5) [21]. The blood glucose levels were measured twice in 2 weeks after the STZ injection to identify mice with levels >300 mg/dL. The mice were considered diabetic with this level and were used in the following experiments.

All the mice were accommodated in well-ventilated cages with aspen wood chip bed-ding, initially grouped six per cage for the first set of experiments and five per cage for subsequent experiments. The mice were maintained under regulated environmental conditions, including a specific-pathogen-free area, 12 h light/12 h dark cycle, room temperature at 21–23 °C, and humidity at 50–60%. They were provided with unrestricted access to standard laboratory food pellets (CLEA Rodent Diet CE-2, CLEA Japan, Tokyo, Japan) and water.

### 4.2. Vaccine Preparation

The recombinant SARS-CoV-2 spike proteins (S-protein, D614G variant and Omicron BA.1 variant) used in this study were prepared by using the silkworm-baculovirus expression vector system (silkworm-BEVS) as previously described [42]. In summary, the S-protein was mutated at the furin cleavage site (682-GSAS-685) and introduced with two proline substitutions (K986P and V987P). In addition, chicken cartilage matrix protein (CMP) was fused to the C-terminus of the S-protein to promote stable trimerization. Then, recombinant baculoviruses expressing the designed S-protein were amplified in cultured silkworm cells and infected with silkworm larvae. The recombinant S-protein was obtained by affinity chromatography from the sera of infected silkworm larvae and stored at −80 °C in PBS supplemented with 10% trehalose until use. A mixture of 5 µg of D614G variant and Omicron BA.1 variant S-protein mixed with an equivalent volume of adjuvant was administered by intramuscular injection to the mice.

### 4.3. Study Design

For the first set of experiments, 24 BALB/c mice were randomly divided into 4 groups according to the received antigen: Alum (Alhydrogel^®^ 2%, Cat# vac-alu-10, InvivoGen, San Diego, CA, USA), MF-59 (AssaVax^™^, Cat# vac-adx-10, InvivoGen, San Diego, CA, USA), Alum + Lipid A (Cat# 24018-s, Peptide Institute, Inc., Osaka, Japan), and Alum + MPL (MPLA-SM, Cat# tlrl-mpla2, InvivoGen, San Diego, CA, USA). Each group was administered with the S-protein mixture/adjuvant (60 μL per mouse) by intramuscular injection followed by a booster dose after two weeks. The echocardiography was performed at 3 weeks, the serum antibody titer was measured at 3 and 4 weeks, and the sample collection was at 4 weeks.

For the second set of experiments, the mice were randomly divided into six groups, with five mice in each group: WT + saline, WT + vaccine, ob/ob + saline, ob/ob + vaccine, STZ + saline, and STZ + vaccine. The vaccinated groups were administered an S-protein mixture injection followed by a booster dose after two weeks, while the non-vaccinated mice received an equivalent volume of saline in both doses.

At the age of 8 weeks, blood glucose measurement, echocardiography, and serum antibody titer were carried out as the basal measurements before vaccination. The measurements were taken again at every other week after the 2nd dose of the vaccine. After 10 weeks, the mice were euthanized, blood samples were collected from the orbital sinus into a 1.5 mL tube, and then the serum was separated. The body organs were collected, washed with PBS and weighed, and used later for further analysis.

### 4.4. Enzyme-Linked Immunosorbent Assay (ELISA)

Serum IL-6 and IL-1β were evaluated using the DuoSet™ commercial kit (Cat# DY 406-05 and Cat# DY 401-05, R&D Systems, Minneapolis, MN, USA) according to the manufacturer manual. Serum insulin was detected using the Mouse/Rat Insulin Measurement Kit (Cat# M1108, Morinaga, Kanagawa, Japan) according to the manufacturer manual. The S-protein IgG antibody titer was determined using the enzyme-linked immunosorbent assay (ELISA) method as follows: A coating buffer containing the S-protein antigen to a 96-well plate, which was then left to incubate overnight at a temperature of 4 °C. The following day, the wells were cleaned using PBS/T. Subsequently, the wells were blocked using 10% FBS for one hour. After another round of washing with PBS/T, the samples were introduced into the wells and allowed to incubate for an hour at room temperature. After the initial steps, the wells were once again cleaned using PBS/T. Following this, a secondary antibody (Goat F(ab) Anti-Mouse IgG (HRP), Cat# ab6823, Abcam, Tokyo, Japan) was introduced into all the wells and left to incubate for an hour. After another round of washing, TMB (Cat# 00-4201-56, Invitrogen, Vienna, Austria) was added to the wells. Once color development was observed, 1 N H_2_SO_4_ solution was added to stop the reaction. The absorbance was read at 450 nm.

### 4.5. Echocardiography

The mouse was placed on a warm pad and given anesthesia (1–2% isoflurane supplemented with 100% oxygen) before undergoing transthoracic echocardiography. Prospect T1 (S-Sharp Corporation, New Taipei, Taiwan) was used to capture images of the heart’s structure and function in vivo. Two-dimensional guided M-mode images of the left ventricle in parasternal long-axis views at the papillary muscle level were recorded to assess the LVEF and LVFS in conscious experimental animals. Three or more consecutive cardiac cycles were averaged for all the analyses.

### 4.6. Blood Glucose

A blood glucose analysis was performed on all the mice before and every two weeks after vaccine administration. Blood samples were collected from the caudal vein and directly applied on test strips using a glucometer (Glucose Pilot, Cat# 10002 and 10003, Syntron Bioresearch, Inc., Carlsbad, CA, USA). For each mouse, three readings were recorded, and the average measurement was calculated.

### 4.7. Dry Chem Measurements

The total cholesterol (TC), Alanine Transaminase (ALT), and Aspartate Transaminase (AST) were measured by adding 10 µl of serum to Dry chemistry slides (Cat# 471-0551, 474-03341, and 477-03331, respectively, FUJI DRI-CHEM, Tokyo, Japan) and analyzed with a Dry chemistry analyzer (FUJI DRI-CHEM NX500, FUJIFILM, Tokyo, Japan).

### 4.8. Histological Analysis

The mouse hearts and spleens were isolated and washed with PBS before embedding in optimal cutting temperature (O.C.T.) compound (Cat# 90501, Sakura Finetek, Torrance, CA, USA) and snap-frozen in a bath of 2-methylbutane with crushed dry ice. The heart and spleen slices were cut on a cryostat to 12 and 9 μm thickness, respectively. The slices were fixed using 4% PFA and stained with HE (hematoxylin and eosin).

Immunohistochemistry for 4% PFA-fixed splenic tissue slices was then carried out. The sections were blocked with 1% BSA (Cat# 015-21274, FUJIFILM Wako Pure Chemical Corporation, Osaka, Japan) and then incubated with primary antibodies CD4 Antibody (Rat IgG) (Cat# 130310, Biolegend, San Diego, CA, USA), CD8a Antibody (Rat IgG) (Cat# 100713, Biolegend, San Diego, CA, USA), and CD45R/B220 Antibody (Rat IgG) (Cat# 103231, Biolegend, San Diego, CA, USA) overnight at 4 °C. The next day, the slides were washed with PBS and incubated with secondary antibodies Alexa Fluor™ 568 goat Anti-Rat IgG (Cat# 2379471, Invitrogen, Carlsbad, CA, USA) and DAPI (Cat# 340-07971, Dojindo, Kumamoto, Japan).

All the sections were photographed using a BZ-X700 microscope (Keyence, Osaka, Japan). ImageJ software (V1.54g, NIH, Stapleton, NY, USA) was used to calculate the myocardial CSA (cross-section area) for each heart section and white pulp area ratio (white pulp area/spleen area) and the CD4-, CD8-, and CD45R/B220-positive area in each spleen section.

### 4.9. Statistics

The results are presented as the means ± S.E.M. Statistical comparisons were carried out with an ordinary one-way ANOVA followed by Tukey’s multiple comparison test (for three and more groups of variables) and a two-way ANOVA followed by Bonferroni multiple comparison test (for three or more groups of variables that change over time). Values of *p* < 0.05 were considered to be statistically significant.

## Data Availability

The original contributions presented in the study are included in the article/Supplementary Materials, further inquiries can be directed to the corresponding author.

## Supplementary Materials

The following supporting information can be downloaded at: https://www.mdpi.com/article/10.3390/ijms251910379/s1.
