# Supplementary material for: Diabetic Mice Spleen Vulnerability Contributes to Decreased Persistence of Antibody Production after SARS-CoV-2 Vaccine
Source: Int J Mol Sci. 2024 Sep 26;25(19):10379. doi: 10.3390/ijms251910379 (PMC11476529; doi:10.3390/ijms251910379)
Supplement: Supplementary file 1 [file ijms-25-10379-s001.zip › ijms-3187587-supplementary.pdf]

Supplementary Information

Diabetic Mice Spleen Vulnerability Contributes to Decreased Persistence of Antibody Production after SARS-CoV-2 Vaccine

Yara Atef [1], Tomoya Ito [1], Akitsu Masuda[2], Yuri Kato [1], Akiyuki Nishimura[3,4,5], Yasunari Kanda[6], Jun Kunisawa[7], Takahiro Kusakabe[8], Motohiro Nishida [1,2,3,4*]

Affiliations:

[1]Department of Physiology, Graduate School of Pharmaceutical Sciences, Kyushu University, 3-1-1 Maidashi, Higashi-ku, Fukuoka 812-8582, Japan

[2]Laboratory of Creative Science for Insect Industries, Kyushu University Graduate School of Bioresource and Bioenvironmental Sciences, 744 Motooka, Nishi-ku, Fukuoka, 819-0395, Japan

[3]National Institute for Physiological Sciences (NIPS), National Institutes of Natural Sciences (NINS), Okazaki 444-8787, Japan

[4]Exploratory Research Center on Life and Living Systems (ExCELLS), National Institutes of Natural Sciences (NINS), Okazaki 444-8787, Japan

[5]Department of Physiological Sciences, School of Life Science, The Graduate University for Advanced Studies (SOKENDAI), Okazaki 444-8787, Japan

[6]Division of Pharmacology, National Institute of Health Sciences, 3-25-26, Tonomachi, Kawasaki-Ku, Kawasaki, 210-9501, Japan

[7]Laboratory of Vaccine Materials and Laboratory of Gut Environmental System, Microbial Research Center for Health and Medicine, National Institutes of Biomedical Innovation, Health and Nutrition, Osaka, Japan

[8]Laboratory of Insect Genome Science, Kyushu University Graduate School of Bioresource and Bioenvironmental Sciences, 744 Motooka, Nishi-ku, Fukuoka, 819-0395, Japan

*Correspondence and requests for materials should be addressed to:
Motohiro Nishida, Ph.D.
E-mail: nishida@phar.kyushu-u.ac.jp

Supplementary information includes:

- Supplementary Figure S1. HE stained whole spleen section.
- Supplementary Figure S2. IHC stained whole spleen section.

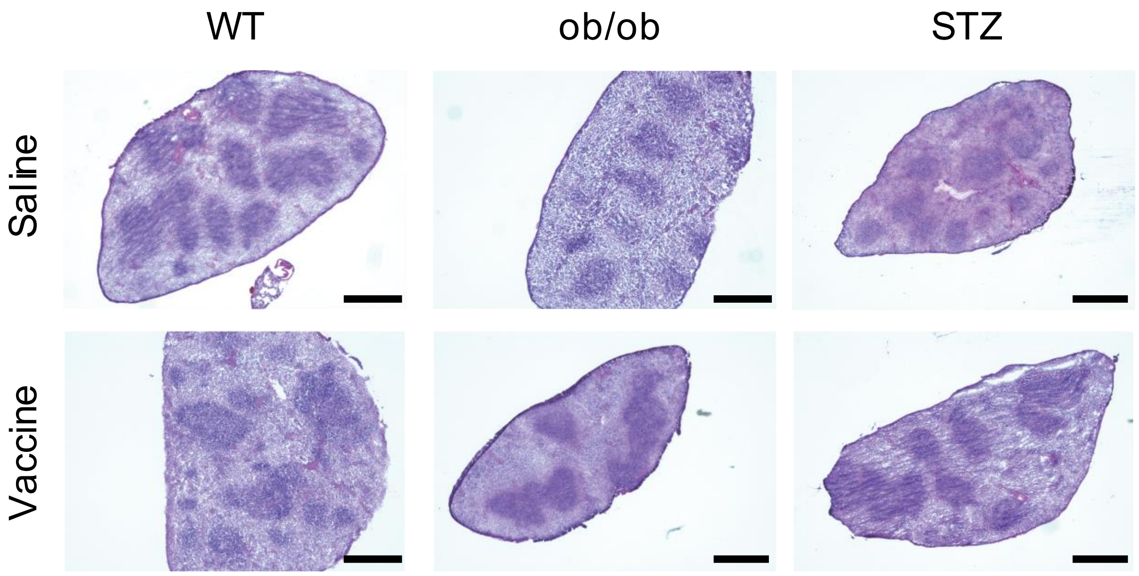

Supplementary Figure S1. HE stained whole spleen section. Representative images of spleen tissue stained with hematoxylin and eosin showing WP (white pulp) and RP (red pulp) regions. Data represent samples from five mice in each group. Scale bar, 500 μm.

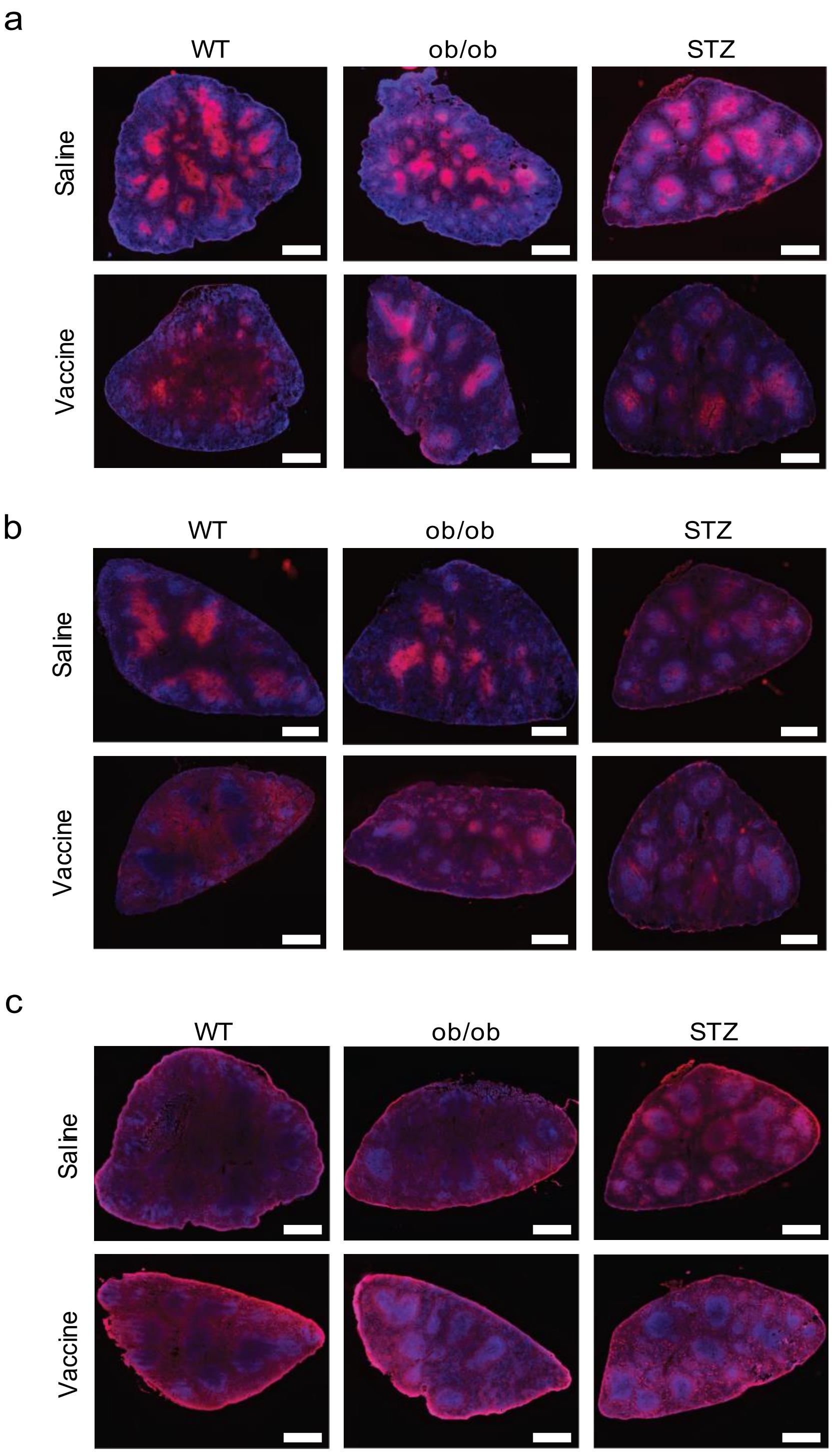

Supplementary Figure S2. IHC stained whole spleen section. (**a**-**c**) Representative images of whole spleen section immunostained with selective antibodies: CD4 for T cells (**a**), CD8 for T cells (**b**) and CD45R/B220 for B cells (**c**). Data represent samples from five mice in each group. Scale bar, 500 μm.
